# Supplementary material for: Mechanical strain promotes skin fibrosis through LRG-1 induction mediated by ELK1 and ERK signalling
Source: Commun Biol. 2019 Oct 4;2:359. doi: 10.1038/s42003-019-0600-6 (PMC6778114; doi:10.1038/s42003-019-0600-6)
Supplement: Supplementary file 1 — Supplementary Materials [file 42003_2019_600_MOESM1_ESM.pdf]

Supplementary Figures:

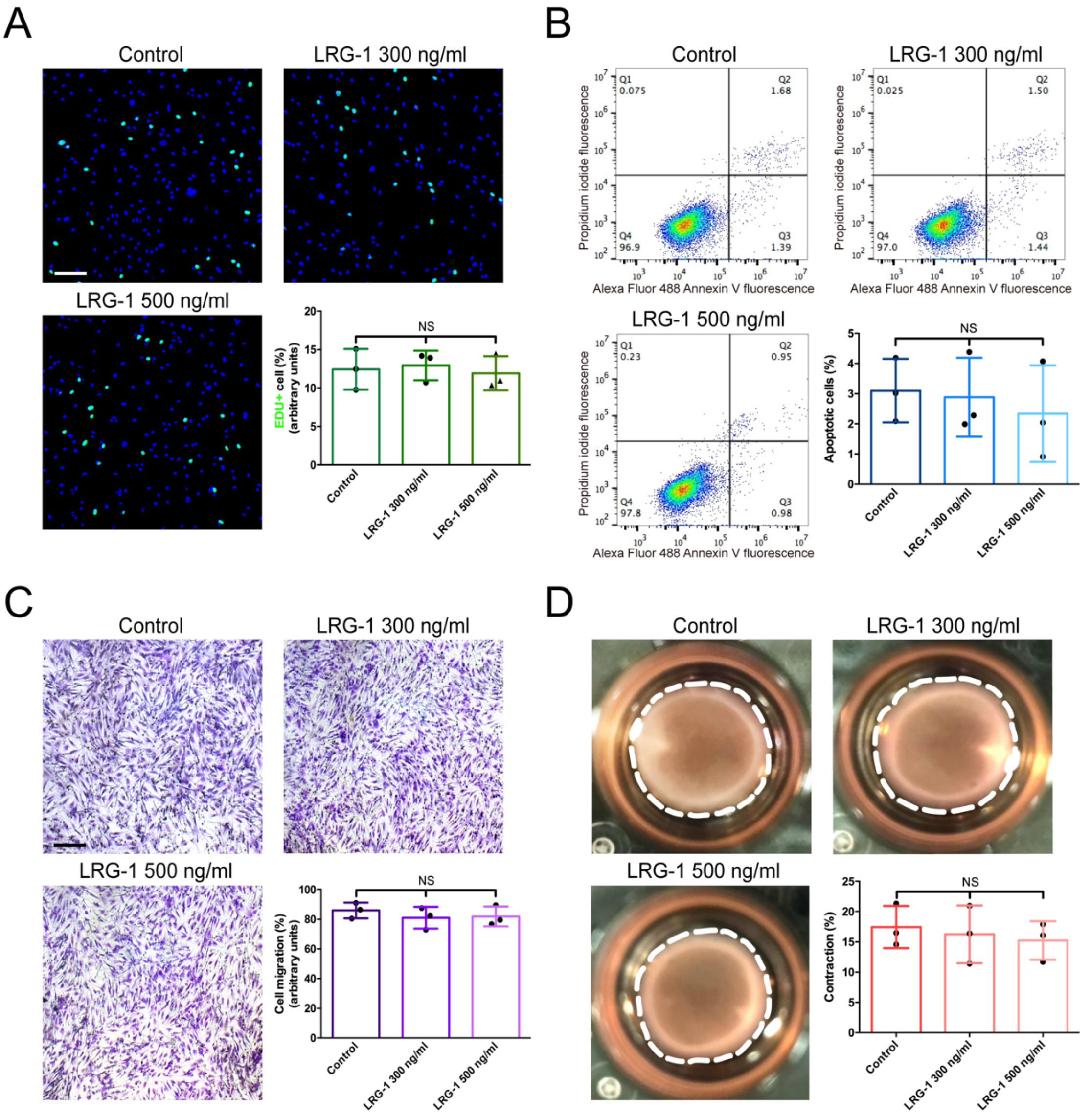

Supplementary Figure 1：LRG-1 did not affect HDFs' proliferation, apoptosis, migration and contraction. (A) EdU(green) proliferation assay was performed 24 hrs after the addition of 300 ng/mL, 500 ng/mL LRG-1 and the control group. DAPI (blue) stains nuclei. (Scale bar = 100 μm). (B) Apoptosis was detected after treating HDFs with LRG-1 for 3 days by flow cytometer. (C) Transwell assay images and quantitative analysis for the migration of HDFs after incubating with different concentrations of LRG-1. (Scale bar = 100 μm). (D) Images and quantification of collagen gel contraction assay captured at time 3 days after LRG-1 treatment.

Dash lines indicate the areas of collagen gel. Data are presented as mean ± SD. NS = not significant. n=3 independent experiments.

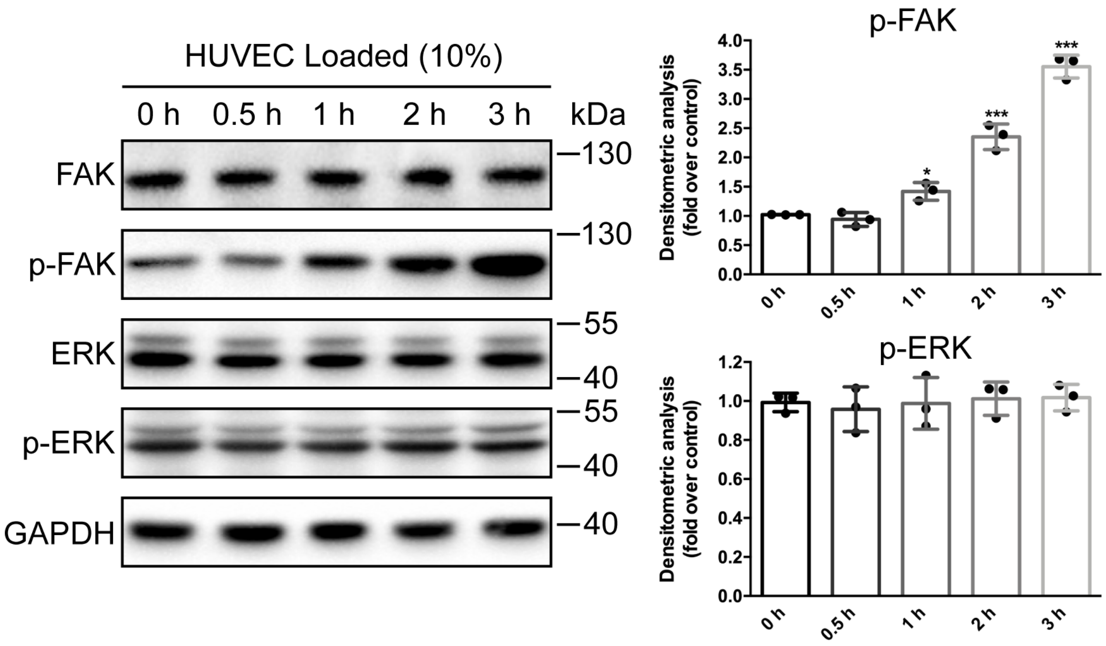

Supplementary Figure 2: Mechanic loading applied to HUVECs. The levels of different loading time related FAK, p-FAK, ERK, p-ERK protein level were measured using Western blotting. Data are presented as mean ± SD. *P < 0.05, ***P < 0.001. n=3 independent experiments.

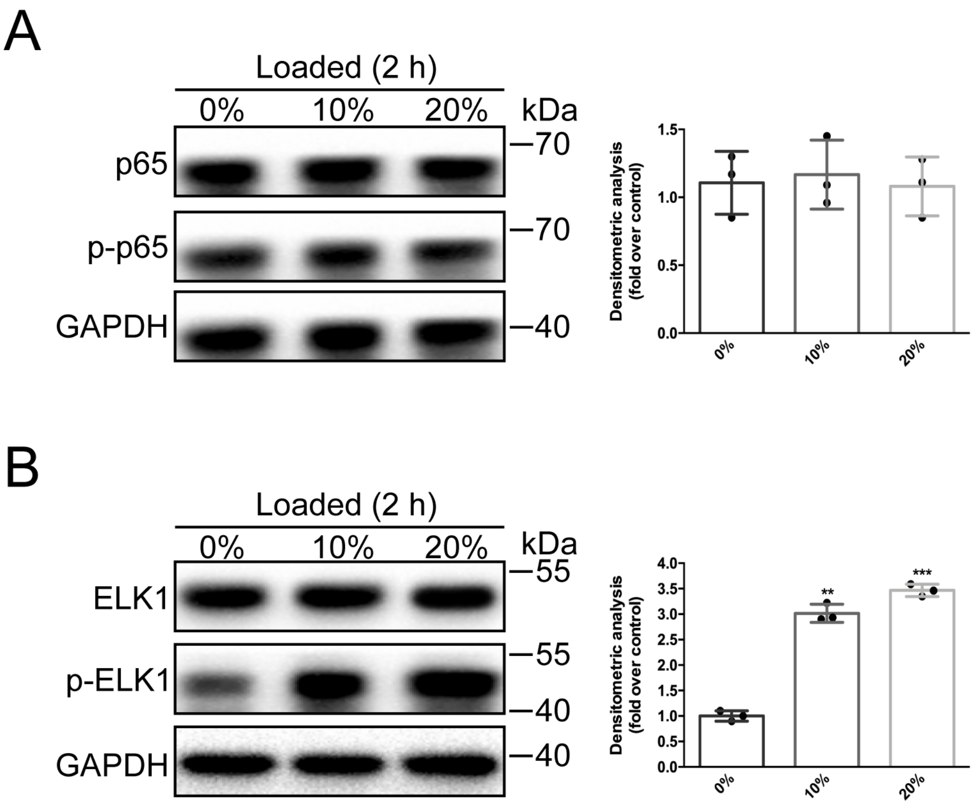

Supplementary Figure 3: Mechanic loading applied to HDFs. The levels of different loading strenth related p65, p-p65, ELK1, p-ELK1 protein level were measured using Western blotting. Data are presented as mean ± SD. NS = not significant, **P < 0.01, ***P < 0.001. n=3 independent experiments.

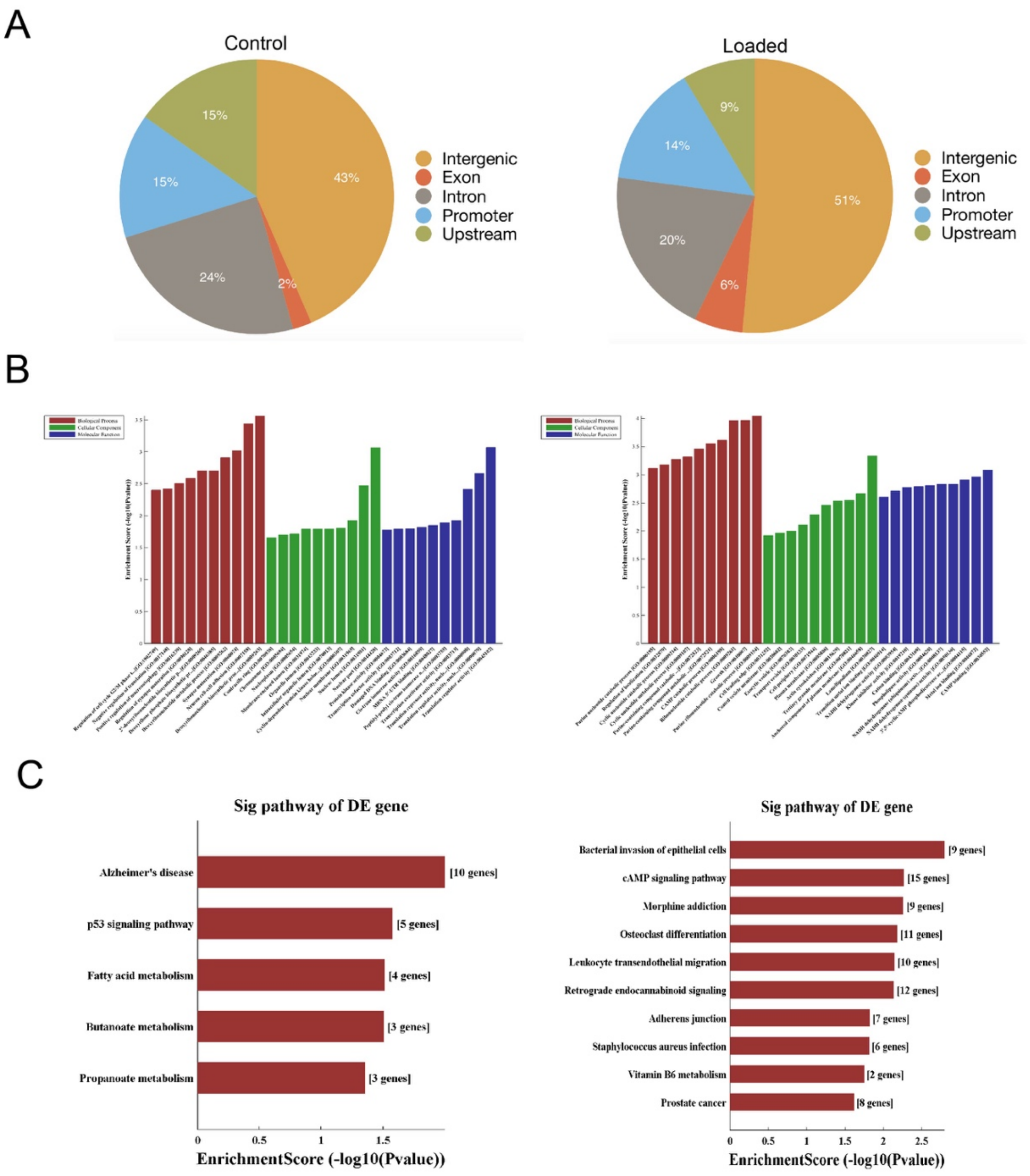

Supplementary Figure 4: ELK1 ChIP-seq analysis. (A) Relationship of genome-wide ELK1 binding sites to annotated genes in HDFs loading and control group. (B) GO analysis of ChIP-seq results. The bar plot shows the top ten Enrichment Score value of the significant enrichment terms. (C) Pathway analysis of ChIP-seq results using KEGG pathway. Enrichment Score means value of the pathway, equals "-log10 (*P* value)”.

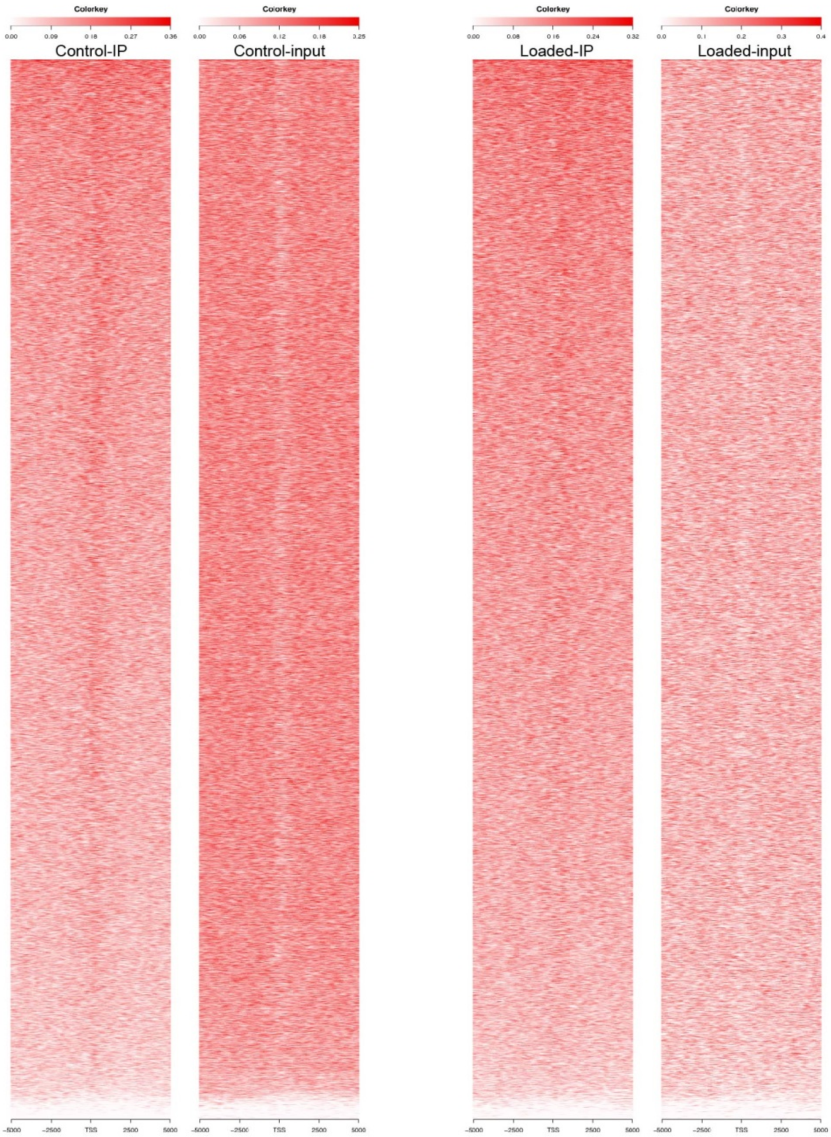

Supplementary Figure 5: Peak annotation in control and loading group. The peaks in samples were annotated by the nearest gene using the newest UCSC database.

A

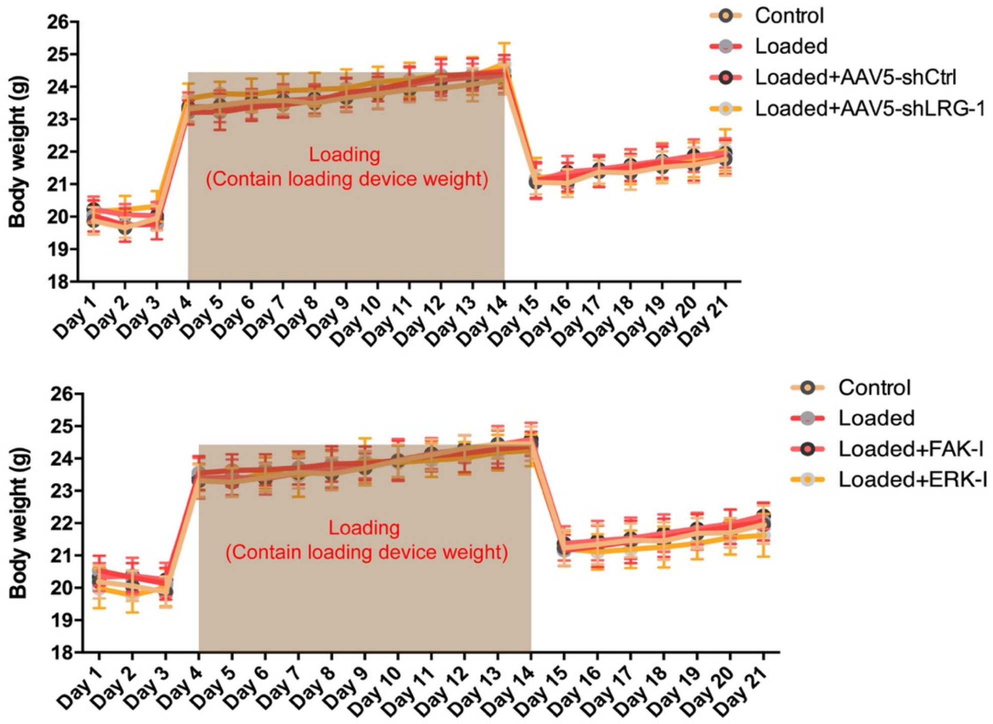

B

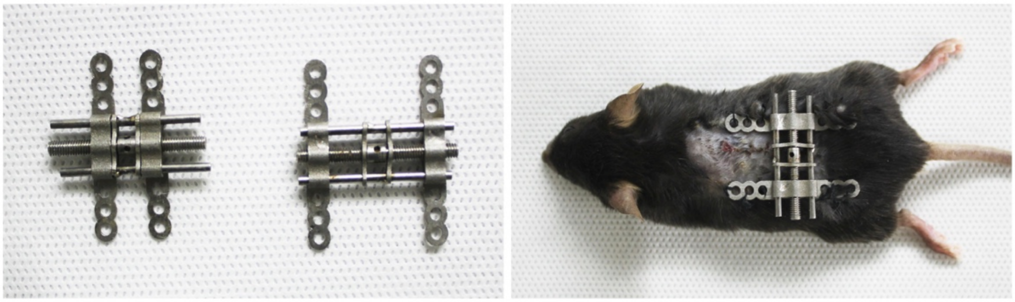

Supplementary Figure 6: Body weight measurement and mechanical loading device. (A) Record of mouse body weight during the experiment. (B) The picture of mechanical loading devices (left) and the representative image of hypertrophic scar model (right).

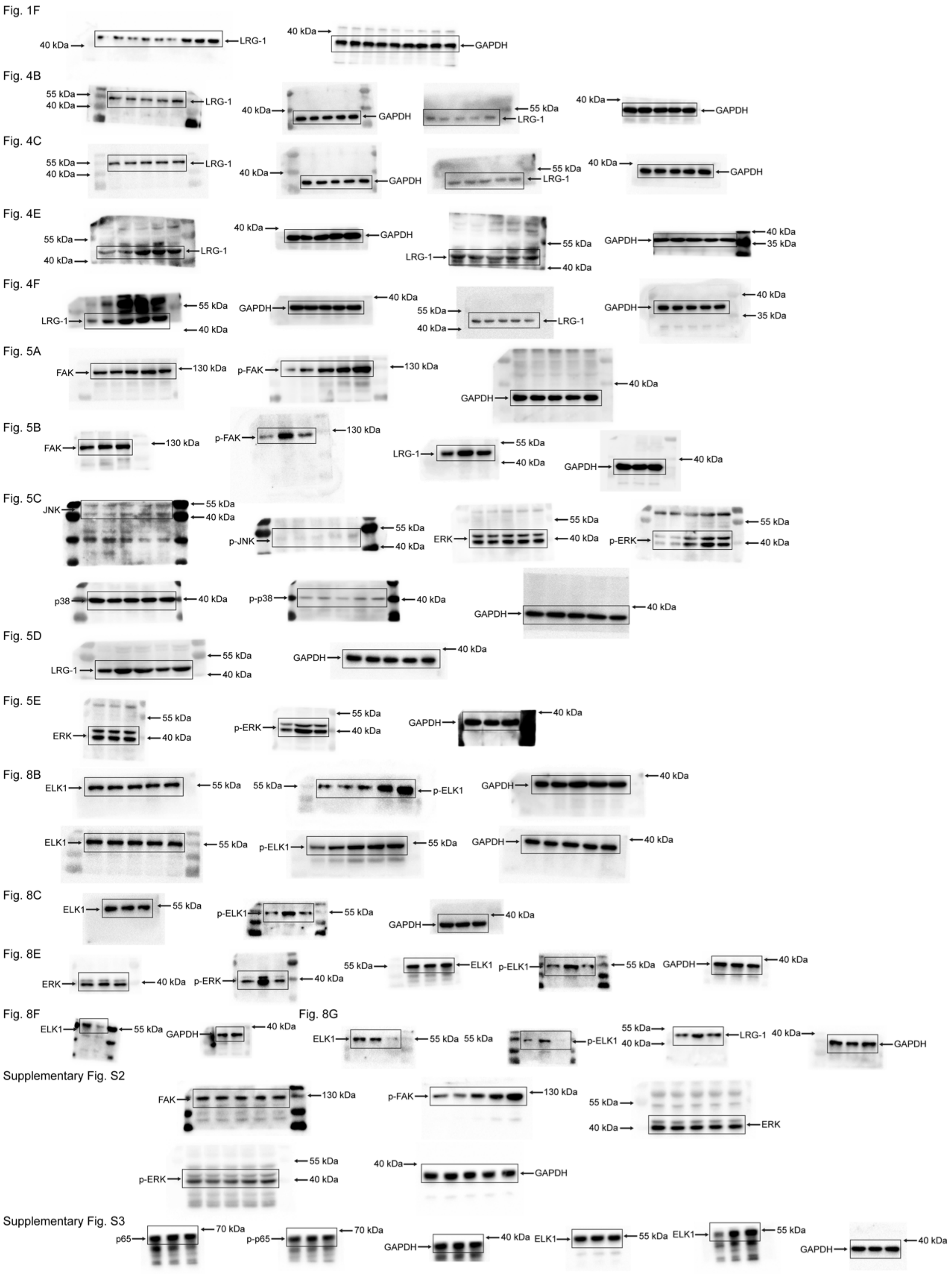

Supplementary Figure 7: Full western blot images.

Supplementary Table 1

Transcription factors predicted by PROMO

AP-2alphaA
AR
C/EBPalpha
C/EBPbeta
c-Ets-1
c-Ets-2
c-Jun
c-Myb
c-Myc
EBF
Elk-1
ER-alpha
FOXP3
GATA-1
GATA-2
GR
GR-alpha
GR-beta
HIF-1
HNF-1A
HNF-3alpha
Ik-1
IRF-1
IRF-2
LEF-1
MAZ
MITF
NF-AT1
NF-AT2
NFI/CTF
NF-kappaB1
NF-Y
p53
Pax-5
PEA3
PITX2
PR A
PR B
PXR-1
RAR-beta
RXR-alpha
STAT1beta
STAT4
T3R-beta1
TBP
TCF-4
TCF-4E
TFIID
TFII-I
USF1
USF2
VDR

WT1 I
WT1 I -KTS
WT1
XBP-1
YY1

Transcription factors predicted by JASPAR

MA0753.1	ZNF740	8.93569	0.856234915	LRG1_promoter	1745	1754	+	GCCCCCCAAA
MA1125.1	ZNF384	16.3083	0.991393329	LRG1_promoter	799	810	+	AAAAAAAAAAAA
MA1154.1	ZNF282	13.9623	0.853242423	LRG1_promoter	1700	1716	-	ACTTCCCCCAACCCGGA
MA0751.1	ZIC4	11.0977	0.865457011	LRG1_promoter	2238	2252	-	GGCTCCCTGCTGCCC
MA0103.3	ZEB1	13.3697	0.983762911	LRG1_promoter	2224	2234	-	CCCACCTGGGC
MA0698.1	ZBTB18	8.78345	0.864590856	LRG1_promoter	831	843	-	ATTACAGGTGTGA
MA0095.1	YY1	8.38313	0.999999988	LRG1_promoter	239	244	+	GCCATC
MA0726.1	VSX2	8.51767	0.897005423	LRG1_promoter	68	75	+	CTAATTTT
MA0725.1	VSX1	8.50137	0.907910903	LRG1_promoter	68	75	+	CTAATTTT
MA0724.1	VENTX	10.5722	0.941678823	LRG1_promoter	281	289	-	ATCCATTAA
MA0693.2	VDR	11.8015	0.999999997	LRG1_promoter	132	139	-	TGAGTTCA
MA0093.2	USF1	13.0047	0.944049386	LRG1_promoter	873	883	-	CTCATGTGATC
MA0721.1	UNCX	8.16629	0.91830657	LRG1_promoter	507	514	+	TAAATTAG
MA1123.1	TWIST1	8.46196	0.853486669	LRG1_promoter	831	843	-	ATTACAGGTGTGA
MA0692.1	TFEB	11.637	0.932187354	LRG1_promoter	683	692	+	GGCACGTGCC
MA0831.1	TFE3	11.3067	0.940485061	LRG1_promoter	683	692	+	GGCACGTGCC
MA0691.1	TFAP4	8.07417	0.858186433	LRG1_promoter	136	145	-	ATCACCTGAG
MA0814.1	TFAP2C	13.9544	0.971672697	LRG1_promoter	2354	2364	+	AGCCCCAGGCT
MA0812.1	TFAP2B	14.7287	0.973472412	LRG1_promoter	2354	2364	+	AGCCCCAGGCT
MA0003.3	TFAP2A	14.1092	0.9765939	LRG1_promoter	2354	2364	+	AGCCCCAGGCT
MA0809.1	TEAD4	11.7869	0.971929672	LRG1_promoter	2103	2112	+	GACATTCCAG
MA0808.1	TEAD3	13.4105	0.999999999	LRG1_promoter	2104	2111	+	ACATTCCA
MA1121.1	TEAD2	13.2113	0.930732708	LRG1_promoter	2102	2114	+	GGACATTCCAGCT
MA0090.1	TEAD1	11.4254	0.881766848	LRG1_promoter	2103	2114	+	GACATTCCAGCT
MA0830.1	TCF4	13.3605	0.991762451	LRG1_promoter	832	841	+	CACACCTGTA
MA0807.1	TBX5	12.8551	0.99999999	LRG1_promoter	831	838	-	AGGTGTGA
MA0806.1	TBX4	13.6527	1.000000002	LRG1_promoter	831	838	-	AGGTGTGA
MA0690.1	TBX21	10.6349	0.888211575	LRG1_promoter	830	839	-	CAGGTGTGAG
MA0689.1	TBX20	9.68513	0.856431885	LRG1_promoter	1147	1157	-	TAGGTATGATG
MA0688.1	TBX2	10.5443	0.894317743	LRG1_promoter	829	839	-	CAGGTGTGAGC
MA0803.1	TBX15	15.2832	1.000000001	LRG1_promoter	831	838	-	AGGTGTGA
MA0805.1	TBX1	15.051	1.000000002	LRG1_promoter	831	838	-	AGGTGTGA
MA0802.1	TBR1	11.3358	0.907119626	LRG1_promoter	829	838	-	AGGTGTGAGC
MA0084.1	SRY	10.2339	0.952189815	LRG1_promoter	1280	1288	+	GGAAACAAT
MA0828.1	SREBF2	10.1642	0.896643448	LRG1_promoter	136	145	-	ATCACCTGAG
MA0687.1	SPIC	15.8326	0.92949462	LRG1_promoter	1265	1278	+	GAAATGAGGAAGAA
MA0081.1	SPIB	10.4696	1.000000002	LRG1_promoter	328	334	-	AGAGGAA
MA0080.4	SPI1	11.9028	0.869545074	LRG1_promoter	1265	1278	+	GAAATGAGGAAGAA
MA0747.1	SP8	13.0864	0.922012037	LRG1_promoter	57	68	+	ACCACGCCCCGC
MA0685.1	SP4	10.7994	0.858966414	LRG1_promoter	1849	1865	+	CTGACCCCTCCCCTTCC
MA0746.1	SP3	12.5547	0.935715056	LRG1_promoter	57	67	+	ACCACGCCCCG
MA0079.3	SP1	14.4264	0.962641815	LRG1_promoter	1905	1915	-	TCCCCACCCCC
MA0077.1	SOX9	12.0334	0.972444827	LRG1_promoter	1282	1290	-	CTATTGTTT
MA1152.1	SOX15	12.3627	0.9756902	LRG1_promoter	1281	1290	-	CTATTGTTTC
MA1120.1	SOX13	11.1946	0.937163446	LRG1_promoter	1282	1292	+	AAACAATAGCA
MA0442.2	SOX10	11.4982	0.940409456	LRG1_promoter	1407	1417	+	GTCACAAAGCA
MA0745.1	SNAI2	11.9049	0.983983179	LRG1_promoter	833	841	-	TACAGGTGT
MA0630.1	SHOX	8.36345	0.921822463	LRG1_promoter	507	514	-	CTAATTTA
MA0856.1	RXRG	18.2229	0.939818484	LRG1_promoter	1688	1701	-	GAGTTCAAGGGTCA
MA0855.1	RXRB	17.305	0.918275293	LRG1_promoter	1688	1701	-	GAGTTCAAGGGTCA
MA0002.1	RUNX1	8.88468	0.859560533	LRG1_promoter	1619	1629	+	GTTTATGGTAA
MA1151.1	RORC	10.791	0.911093001	LRG1_promoter	223	234	+	TTAAGTGGGACA
MA1150.1	RORB	9.03068	0.87267364	LRG1_promoter	265	275	-	AATGAGATCAC
MA0072.1	RORA	11.6443	0.86260262	LRG1_promoter	222	235	+	TTTAAGTGGGACAA
MA0719.1	RHOXF	9.36542	0.984800509	LRG1_promoter	173	180	-	GTAATCCC
MA0799.1	RFX4	9.37364	0.854245348	LRG1_promoter	693	708	-	AGTAGCTGGGACTACG
MA1117.1	RELB	10.4074	0.892063077	LRG1_promoter	1909	1919	-	GAAGTCCCCAC
MA0101.1	REL	10.5207	0.925128657	LRG1_promoter	1911	1920	+	GGGGACTTCC

MA1116.1	RBPJ	11.6043	0.966071619	LRG1_promoter	538	547	-	GCTGGGAATA
MA0718.1	RAX	8.77755	0.906700972	LRG1_promoter	943	952	+	AAAAATTAAC
MA1149.1	RARA::	15.2582	0.910337153	LRG1_promoter	1795	1812	+	AGGGTCAAAAGGGGTTG
MA0716.1	PRRX1	8.00653	0.908591079	LRG1_promoter	507	514	+	TAAATTAG
MA0065.1	PPARG:	16.1735	0.857580846	LRG1_promoter	1685	1704	-	CCGGAGTTCAAGGGTCAA
MA0792.1	POU5F1	9.31226	0.886971921	LRG1_promoter	1554	1562	-	TATGTTGAT
MA1115.1	POU5F1	9.26916	0.872375264	LRG1_promoter	72	82	-	AAATACAAAAA
MA0789.1	POU3F4	8.52894	0.874863764	LRG1_promoter	1554	1562	-	TATGTTGAT
MA0786.1	POU3F1	9.89489	0.861451369	LRG1_promoter	1621	1632	+	TTATGGTAAATC
MA0785.1	POU2F1	9.71651	0.866292397	LRG1_promoter	1620	1631	+	TTTATGGTAAAT
MA0714.1	PITX3	9.89942	0.932287049	LRG1_promoter	173	181	-	TGTAATCCC
MA0713.1	PHOX2A	9.74382	0.874024149	LRG1_promoter	610	620	-	TGATGCAATTT
MA0014.2	PAX5	13.9988	0.891110229	LRG1_promoter	1070	1088	+	CTAGGCAACAGTGCAAGA
MA0068.2	PAX4	8.71752	0.887966902	LRG1_promoter	507	514	+	TAAATTAG
MA0712.1	OTX2	10.0122	0.952199566	LRG1_promoter	1843	1850	+	GTAATCCT
MA0711.1	OTX1	10.1437	0.957088898	LRG1_promoter	173	180	-	GTAATCCC
MA0757.1	ONECU	12.4353	0.873602307	LRG1_promoter	1543	1556	+	AAAAAAGCAATATC
MA0756.1	ONECU	12.0759	0.870708809	LRG1_promoter	1543	1556	+	AAAAAAGCAATATC
MA0679.1	ONECU	11.017	0.87436901	LRG1_promoter	1543	1556	+	AAAAAAGCAATATC
MA0842.1	NRL	9.56025	0.907197015	LRG1_promoter	786	796	+	AAAATGCTGTC
MA0160.1	NR4A2	11.6387	0.999999991	LRG1_promoter	1404	1411	+	AAGGTCAC
MA1112.1	NR4A1	14.6797	0.991048781	LRG1_promoter	1402	1411	+	CAAAGGTCAC
MA1111.1	NR2F2	14.7742	0.992976333	LRG1_promoter	1402	1412	+	CAAAGGTCACA
MA0017.1	NR2F1	15.0579	0.935945613	LRG1_promoter	1688	1701	+	TGACCCTTGAACTC
MA0710.1	NOTO	9.76896	0.94048362	LRG1_promoter	506	515	+	TTAAATTAGC
MA0675.1	NKX6-2	9.14932	0.952498518	LRG1_promoter	281	288	-	TCCATTAA
MA0674.1	NKX6-1	8.89121	0.930065889	LRG1_promoter	281	288	-	TCCATTAA
MA0673.1	NKX2-8	11.3996	0.976671127	LRG1_promoter	222	230	-	CCACTTAAA
MA0672.1	NKX2-3	13.2545	0.983672209	LRG1_promoter	222	231	-	CCCACTTAAA
MA0778.1	NFKB2	16.6654	0.948598822	LRG1_promoter	2348	2360	-	TGGGGCTTCCCCT
MA0105.3	NFKB1	15.1059	0.969346956	LRG1_promoter	1912	1922	+	GGGACTTCCCC
MA0671.1	NFIX	10.2337	0.986432629	LRG1_promoter	1397	1405	+	CTTGCCAAA
MA0025.1	NFIL3	10.066	0.870475111	LRG1_promoter	1839	1849	+	TTTTGTAATCC
MA0161.1	NFIC	9.69742	1.000000005	LRG1_promoter	1399	1404	-	TTGGCA
MA0670.1	NFIA	11.5059	0.969313226	LRG1_promoter	1397	1406	+	CTTGCCAAAG
MA0150.1	NFE2L2	10.8814	0.872166933	LRG1_promoter	298	308	-	GTGACACAGTA
MA0625.1	NFATC	11.7047	0.956373971	LRG1_promoter	1277	1286	-	TGTTTCCATT
MA0152.1	NFATC	9.04446	0.914608949	LRG1_promoter	1824	1830	+	ATTTCCA
MA0668.1	NEURO	10.9245	0.943288892	LRG1_promoter	908	917	+	GCCACATGGC
MA1109.1	NEURO	8.82388	0.854593362	LRG1_promoter	907	919	+	GGCCACATGGCAA
MA0057.1	MZF1	9.96082	0.936168088	LRG1_promoter	2346	2355	+	GGAGGGGAAG
MA0104.4	MYCN	11.4169	0.903071543	LRG1_promoter	907	918	+	GGCCACATGGCA
MA1108.1	MXI1	11.9869	0.920507031	LRG1_promoter	681	693	+	GTGGCACGTGCCC
MA0665.1	MSC	10.1069	0.855926505	LRG1_promoter	1216	1225	-	AACAGCTTTG
MA0707.1	MNX1	8.98524	0.915065903	LRG1_promoter	280	289	-	ATCCATTAAA
MA0825.1	MNT	12.6261	0.976491841	LRG1_promoter	683	692	+	GGCACGTGCC
MA0664.1	MLXIPL	10.6342	0.895148583	LRG1_promoter	683	692	+	GGCACGTGCC
MA0662.1	MIXL1	8.54078	0.927493785	LRG1_promoter	506	515	+	TTAAATTAGC
MA0620.2	MITF	13.0275	0.892495779	LRG1_promoter	870	887	+	GTGGATCACATGAGCTCA
MA0801.1	MGA	14.1739	1	LRG1_promoter	831	838	-	AGGTGTGA
MA0706.1	MEOX2	8.0406	0.892319869	LRG1_promoter	1423	1432	-	TGTCATGAAC
MA0775.1	MEIS3	9.78981	0.945840935	LRG1_promoter	1427	1434	+	ATGACAGA
MA0774.1	MEIS2	9.58515	0.91393997	LRG1_promoter	1427	1434	+	ATGACAGA
MA0773.1	MEF2D	12.6321	0.917390414	LRG1_promoter	76	87	-	ACTAAAAATACA
MA0660.1	MEF2B	12.3772	0.90419093	LRG1_promoter	76	87	-	ACTAAAAATACA
MA0052.2	MEF2A	13.4611	0.90907109	LRG1_promoter	74	88	-	TACTAAAAATACAAA
MA0058.2	MAX	12.7553	0.954084756	LRG1_promoter	527	536	-	AGGCACATGC
MA0703.1	LMX1B	8.20781	0.91472936	LRG1_promoter	944	951	+	AAAATTAA

MA0702.1	LMX1A	8.64574	0.897686688	LRG1_promoter	344	351	+	TTGATTAA
MA0658.1	LHX6	9.12777	0.904902169	LRG1_promoter	343	352	-	CTTAATCAAC
MA0618.1	LBX1	9.24588	0.920429208	LRG1_promoter	1258	1265	+	TTAATAGG
MA1107.1	KLF9	12.8876	0.895594703	LRG1_promoter	2202	2214	+	ACACACACACACC
MA0039.3	KLF4	10.9102	0.900379132	LRG1_promoter	2208	2218	+	ACACACCCCTA
MA0741.1	KLF16	12.0173	0.907390493	LRG1_promoter	1905	1915	-	TCCCCACCCCC
MA0740.1	KLF14	11.5571	0.874738659	LRG1_promoter	56	69	+	CACCACGCCCCGCT
MA1132.1	JUN::JU	8.26125	0.853186939	LRG1_promoter	1389	1398	-	AGTGACTTAA
MA0655.1	JDP2	9.06771	0.876824312	LRG1_promoter	1501	1509	+	ATTACTCAC
MA0050.2	IRF1	14.7515	0.852106275	LRG1_promoter	1097	1117	-	TTTTTTTTTCTTTTTTTTTT
MA0155.1	INSM1	10.8417	0.871441149	LRG1_promoter	2273	2284	+	GGCCTGGGGCCA
MA0824.1	ID4	12.5818	0.966337804	LRG1_promoter	832	841	+	CACACCTGTA
MA0158.1	HOXA5	9.44083	0.985374461	LRG1_promoter	66	73	+	CGCTAATT
MA0650.1	HOXA1:	11.5063	0.920245631	LRG1_promoter	930	939	+	CTATTAAAAA
MA0043.1	HLF	10.0011	0.886303016	LRG1_promoter	1139	1150	+	GGTGACACCATC
MA1106.1	HIF1A	12.7433	0.992172044	LRG1_promoter	684	693	+	GCACGTGCCC
MA0738.1	HIC2	12.1895	0.999999993	LRG1_promoter	2229	2237	-	ATGCCCACC
MA0649.1	HEY2	15.5375	0.998691908	LRG1_promoter	683	692	+	GGCACGTGCC
MA0823.1	HEY1	15.6081	0.996505575	LRG1_promoter	683	692	+	GGCACGTGCC
MA0822.1	HES7	17.3236	0.950348781	LRG1_promoter	682	693	-	GGGCACGTGCCA
MA0821.1	HES5	17.9697	0.967636695	LRG1_promoter	682	693	+	TGGCACGTGCCC
MA0648.1	GSC	8.98412	0.900849441	LRG1_promoter	172	181	-	TGTAATCCCA
MA0646.1	GCM1	8.15524	0.857123589	LRG1_promoter	688	698	-	ACTACGGGCAC
MA0766.1	GATA5	10.9309	0.990764543	LRG1_promoter	2169	2176	-	AGATAAGG
MA0037.2	GATA3	13.3741	0.979436859	LRG1_promoter	2169	2176	-	AGATAAGG
MA0036.2	GATA2	14.6001	0.950651068	LRG1_promoter	2165	2178	+	TCAACCTTATCTGT
MA0849.1	FOXO6	8.40093	0.916861502	LRG1_promoter	342	348	-	ATCAACA
MA0848.1	FOXO4	8.95918	0.925776009	LRG1_promoter	342	348	-	ATCAACA
MA0157.1	FOXO3	9.76145	0.920031709	LRG1_promoter	1279	1286	+	TGGAAACA
MA0033.2	FOXL1	9.02583	0.940661639	LRG1_promoter	342	348	-	ATCAACA
MA0852.2	FOXK1	9.79872	0.85400283	LRG1_promoter	1277	1290	+	AATGGAAACAATAG
MA0031.1	FOXD1	8.10185	0.851736315	LRG1_promoter	1280	1287	+	GGAAACAA
MA0846.1	FOXC2	12.7971	0.933177996	LRG1_promoter	1551	1562	+	AATATCAACATA
MA0032.2	FOXC1	12.6176	0.942894277	LRG1_promoter	1551	1561	+	AATATCAACAT
MA0845.1	FOXB1	11.7535	0.909239998	LRG1_promoter	1551	1561	+	AATATCAACAT
MA1144.1	FOSL2::	9.71132	0.885596233	LRG1_promoter	1501	1510	-	AGTGAGTAAT
MA1138.1	FOSL2::	10.3041	0.899130883	LRG1_promoter	1501	1510	-	AGTGAGTAAT
MA1131.1	FOSL2::	10.0239	0.86526877	LRG1_promoter	1139	1149	+	GGTGACACCAT
MA1143.1	FOSL1::	11.0303	0.927987554	LRG1_promoter	1388	1397	-	GTGACTTAAC
MA1137.1	FOSL1::	10.1072	0.878920457	LRG1_promoter	1499	1511	-	TAGTGAGTAATGC
MA1128.1	FOSL1::	9.05009	0.861040868	LRG1_promoter	1499	1511	+	GCATTACTCACTA
MA1135.1	FOSB::J	10.1645	0.897277602	LRG1_promoter	1501	1510	-	AGTGAGTAAT
MA1141.1	FOS::JU	9.11218	0.85860327	LRG1_promoter	1499	1511	-	TAGTGAGTAATGC
MA0099.3	FOS::JU	9.37784	0.879492238	LRG1_promoter	1501	1510	+	ATTACTCACT
MA0820.1	FIGLA	11.8555	0.95230967	LRG1_promoter	832	841	+	CACACCTGTA
MA0156.1	FEV	9.66612	0.916753867	LRG1_promoter	1262	1269	+	TAGGAAAT
MA0645.1	ETV6	10.9512	0.915530053	LRG1_promoter	1708	1717	+	GGGGGAAGTG
MA0764.1	ETV4	9.1967	0.885223485	LRG1_promoter	1814	1823	+	ACAGGAAACA
MA0762.1	ETV2	13.5087	0.927907693	LRG1_promoter	1813	1823	+	AACAGGAAACA
MA0761.1	ETV1	8.99171	0.881817666	LRG1_promoter	1814	1823	+	ACAGGAAACA
MA0098.3	ETS1	10.7636	0.914858384	LRG1_promoter	1814	1823	+	ACAGGAAACA
MA0644.1	ESX1	8.15005	0.901091703	LRG1_promoter	943	952	+	AAAAATTAAC
MA0258.2	ESR2	12.265	0.891797325	LRG1_promoter	2279	2293	-	TGGTCACTGTGGCCC
MA0760.1	ERF	9.68849	0.870113621	LRG1_promoter	1261	1270	+	ATAGGAAATG
MA0800.1	EOMES	10.0683	0.863261042	LRG1_promoter	827	839	-	CAGGTGTGAGCCA
MA0642.1	EN2	8.22442	0.903611656	LRG1_promoter	343	352	-	CTTAATCAAC
MA0076.2	ELK4	9.74171	0.892290157	LRG1_promoter	1828	1838	-	TTGCTTCCTGG
MA0028.1	ELK1	8.50762	0.894965835	LRG1_promoter	1812	1821	+	GAACAGGAAA

MA0162.2	EGR1	14.7414	0.938048403	LRG1_promoter	1902	1915	-	TCCCCACCCCCGTC
MA0154.2	EBF1	12.7936	0.953273824	LRG1_promoter	1917	1927	+	TTCCCCAGGGT
MA0471.1	E2F6	10.565	0.916895236	LRG1_promoter	999	1009	+	AGGTGGGAGAA
MA0468.1	DUX4	10.7993	0.923253828	LRG1_promoter	610	620	+	AAATTGCATCA
MA0755.1	CUX2	8.33097	0.867349571	LRG1_promoter	105	114	-	TGACCAATAT
MA0819.1	CLOCK	10.3405	0.89730867	LRG1_promoter	683	692	+	GGCACGTGCC
MA0637.1	CENPB	11.9641	0.893997063	LRG1_promoter	217	231	-	CCCACTTAAAACAAA
MA0837.1	CEBPE	10.1752	0.9152068	LRG1_promoter	1838	1847	-	ATTACAAAAT
MA0836.1	CEBPD	9.97164	0.91257998	LRG1_promoter	1838	1847	-	ATTACAAAAT
MA0466.1	CEBPB	15.4494	0.981882518	LRG1_promoter	750	760	+	GATTGCACCAC
MA0102.3	CEBPA	14.4314	0.970878303	LRG1_promoter	751	761	+	ATTGCACCACA
MA0465.1	CDX2	9.56744	0.896741962	LRG1_promoter	1619	1629	-	TTACCATAAAC
MA0636.1	BHLHE4	13.9529	0.949479391	LRG1_promoter	683	692	+	GGCACGTGCC
MA0464.2	BHLHE4	14.3162	0.985731502	LRG1_promoter	683	692	+	GGCACGTGCC
MA0833.1	ATF4	11.8678	0.886669617	LRG1_promoter	96	108	-	ATATGATGAAACC
MA0259.1	ARNT::I	10.9868	0.993668536	LRG1_promoter	684	691	+	GCACGTGC
MA0007.2	AR	8.15376	0.852018636	LRG1_promoter	2053	2067	-	TAGGACAGGTAGTGT
MA0634.1	ALX3	8.95261	0.909388827	LRG1_promoter	506	515	+	TTAAATTAGC
