# Supplementary material for: Mechanical strain promotes skin fibrosis through LRG-1 induction mediated by ELK1 and ERK signalling
Source: Commun Biol. 2019 Oct 4;2:359. doi: 10.1038/s42003-019-0600-6 (PMC6778114; doi:10.1038/s42003-019-0600-6)
Supplement: Supplementary file 3 — Reporting Summary [file 42003_2019_600_MOESM3_ESM.pdf]

nature research

Corresponding author(s): Yifan Zhang&Qingfeng Li

Last updated by author(s): Aug 22, 2019

Reporting Summary

Nature Research wishes to improve the reproducibility of the work that we publish. This form provides structure for consistency and transparency in reporting. For further information on Nature Research policies, see Authors & Referees and the Editorial Policy Checklist.

Statistics

For all statistical analyses, confirm that the following items are present in the figure legend, table legend, main text, or Methods section.

n/a	Confirmed	
☐	☒	The exact sample size (*n*) for each experimental group/condition, given as a discrete number and unit of measurement
☐	☒	A statement on whether measurements were taken from distinct samples or whether the same sample was measured repeatedly
☐	☒	The statistical test(s) used AND whether they are one- or two-sided<br>*Only common tests should be described solely by name; describe more complex techniques in the Methods section.*
☒	☐	A description of all covariates tested
☐	☒	A description of any assumptions or corrections, such as tests of normality and adjustment for multiple comparisons
☒	☐	A full description of the statistical parameters including central tendency (e.g. means) or other basic estimates (e.g. regression coefficient) AND variation (e.g. standard deviation) or associated estimates of uncertainty (e.g. confidence intervals)
☒	☐	For null hypothesis testing, the test statistic (e.g. *F*, *t*, *r*) with confidence intervals, effect sizes, degrees of freedom and *P* value noted<br>*Give P values as exact values whenever suitable.*
☒	☐	For Bayesian analysis, information on the choice of priors and Markov chain Monte Carlo settings
☒	☐	For hierarchical and complex designs, identification of the appropriate level for tests and full reporting of outcomes
☒	☐	Estimates of effect sizes (e.g. Cohen's *d*, Pearson's *r*), indicating how they were calculated

Our web collection on statistics for biologists contains articles on many of the points above.

Software and code

Policy information about availability of computer code

Data collection	no software was used
Data analysis	PROMO, Version 3.0.2; JASPAR, Version 7th release.

For manuscripts utilizing custom algorithms or software that are central to the research but not yet described in published literature, software must be made available to editors/reviewers. We strongly encourage code deposition in a community repository (e.g. GitHub). See the Nature Research guidelines for submitting code & software for further information.

Data

Policy information about availability of data

All manuscripts must include a data availability statement. This statement should provide the following information, where applicable:

- Accession codes, unique identifiers, or web links for publicly available datasets
- A list of figures that have associated raw data
- A description of any restrictions on data availability

ChIP-seq data have been deposited in Gene Expression Omnibus with the primary accession code GSE119433

Field-specific reporting

Please select the one below that is the best fit for your research. If you are not sure, read the appropriate sections before making your selection.

☒ Life sciences ☐ Behavioural & social sciences ☐ Ecological, evolutionary & environmental sciences

For a reference copy of the document with all sections, see nature.com/documents/nr-reporting-summary-flat.pdf

Life sciences study design

All studies must disclose on these points even when the disclosure is negative.

Sample size	10 mice in each group and were repeated 3 times, thus we have totally 30 mice in each group (On day 14, half the mice in each group were sacrificed for the sake of scar harvest and the other half was observed on day 21. The size were determined according to Charan J, Kantharia ND's method (How to calculate sample size in animal studies?. Journal of pharmacology & pharmacotherapeutics. 2013 Oct;4(4):303). According to their formula in this article we calculate that 10 mice in each group and repeated 3 times, totally 30 mice in each group are sufficient.
Data exclusions	no data excluded
Replication	repeated 3 times
Randomization	At first, mice were randomly selected to each group. On day 14, half the mice in each group were randomly selected sacrificed for the sake of scar harvest and the other half was observed on day 21.
Blinding	there is no blinding

Reporting for specific materials, systems and methods

We require information from authors about some types of materials, experimental systems and methods used in many studies. Here, indicate whether each material, system or method listed is relevant to your study. If you are not sure if a list item applies to your research, read the appropriate section before selecting a response.

Materials & experimental systems

n/a	Involved in the study
☐	☒ Antibodies
☐	☒ Eukaryotic cell lines
☒	☐ Palaeontology
☐	☒ Animals and other organisms
☐	☒ Human research participants
☒	☐ Clinical data

Methods

n/a	Involved in the study
☐	☒ ChIP-seq
☒	☐ Flow cytometry
☒	☐ MRI-based neuroimaging

Antibodies

Antibodies used	anti-LRG-1 antibody (abcam, ab178698, 1:5000)， anti-LRG-1 antibody (abcam, ab231188, 1:5000)， anti-FAK antibody (abcam, ab40794,1:1000), anti-FAK (phosphoS732) antibody (abcam, ab4792,1:500), anti-p44/42 MAPK antibody (Erk1/2) (CST, #4695, 1:1000), anti-Phospho-p44/42 MAPK antibody (Erk1/2) (CST, #4370,1:2000), anti-SAPK/JNK antibody (CST, #9252, 1:1000), anti-Phospho-SAPK/JNK antibody (Thr183/Tyr185) (CST, #4668,1:1000), anti-p38 MAPK antibody (CST, #9212,1:1000), anti-Phospho-p38 MAPK antibody (Thr180/Tyr182) (CST, #4511, 1:1000), anti-ELK1 antibody (abcam, ab131465, 1:1000), anti-ELK1 (phosphoS383) antibody (abcam, ab218133, 1:500), anti-GAPDH antibody (CST, #5174, 1:1000)
Validation	all antibodies were validated in the websites and were used in published papers

Eukaryotic cell lines

Policy information about cell lines

Cell line source(s)	HUVECs were purchased from the ATCC (American Type Culture Collection)
Authentication	Cell line authentication is achieved by genetic profiling using polymorphic short tandem repeat (STR) loci.
Mycoplasma contamination	negative for mycoplasma contamination
Commonly misidentified lines (See ICLAC register)	no cell lines used are listed in the ICLAC register

Animals and other organisms

Policy information about studies involving animals; ARRIVE guidelines recommended for reporting animal research

Laboratory animals	C57BL/6 mice which were eight weeks old for the experiment were purchased from Shanghai Slac Laboratory Animal (Slac, Shanghai, China).

Wild animals	did not involve
Field-collected samples	did not involve
Ethics oversight	All procedures were performed in accordance with Guide for the Care and Use of Laboratory Animals which was approved by the Committee on the Ethics of Animal Experiments of Shanghai Jiao Tong University School of Medicine.

Note that full information on the approval of the study protocol must also be provided in the manuscript.

Human research participants

Policy information about studies involving human research participants

Population characteristics	18 to 45 years old Asian male/female diagnosed hypertrophic scar with no treatment before
Recruitment	randomly in plastic operation
Ethics oversight	Normal human skin and HS samples were obtained from Shanghai Ninth People's Hospital with ethics approval from local Human Research Ethics Committee of Shanghai Jiao Tong University School of Medicine in accordance with the Declaration of Helsinki principles. Written informed consent was obtained from patients undergoing surgery to obtain excised tissue.

Note that full information on the approval of the study protocol must also be provided in the manuscript.

ChIP-seq

Data deposition

- [x] Confirm that both raw and final processed data have been deposited in a public database such as GEO.
- [ ] Confirm that you have deposited or provided access to graph files (e.g. BED files) for the called peaks.

Data access links<br>*May remain private before publication.*	Go to https://www.ncbi.nlm.nih.gov/geo/query/acc.cgi?acc=GSE119433<br>Enter token qvalmmwghpyxpur into the box
Files in database submission	HDF-jiali-IP<br>HDF-control-IP<br>HDF-jiali-input<br>HDF-control-input
Genome browser session<br>(e.g. UCSC)	final submission

Methodology

Replicates	assayed in triplicate and repeated at least three times
Sequencing depth	DNA samples were end-repaired, A tailed, and adaptor ligated using TruSeq Nano DNA Sample Prep Kit (#FC-121-4002, Illumina), following the manufacturer's instructions. ~200-1500 bp fragments were size selected using AMPure XP beads. The final size of the library was confirmed by Agilent 2100 Bioanalyzer.
Antibodies	purified anti-ELK1(abcam, ab32106), anti-NFκB p65 (abcam, ab19870) or normal rabbit IgG
Peak calling parameters	MACS v1.4 (Model-based Analysis of ChIP-seq) software was used to detect the peak from ChIP-seq data. We run MACS with mapped reads. An EXCEL/BED format file containing the ChIP-enriched regions was generated for each comparison/sample.
Data quality	Sample Quality Control<br>1 DNA Quality Control<br>The Qubit® Fluorometer is used for accurate measurement of DNA concentration (Quant-iT™ dsDNA High-Sensitivity (HS) Assay Kit, Invitrogen). The concentration of each sample was determined before sequencing library preparation.<br>2 Quality Assessment of Sequencing Library<br>The Agilent 2100 Bioanalyzer is used for accurate assessment the quality and concentration of sequencing library, the size and concentration of each sample was determined after sequencing library preparation.
Software	Sequencing was performed on Illumina HiSeq 4000 using HiSeq 3000/4000 SBS Kit (300 cycles) (#FC-410-1003, Illumina), according to the manufacturer's instructions.
