# Supplementary material for: Mechanical strain promotes skin fibrosis through LRG-1 induction mediated by ELK1 and ERK signalling
Source: Commun Biol. 2019 Oct 4;2:359. doi: 10.1038/s42003-019-0600-6 (PMC6778114; doi:10.1038/s42003-019-0600-6)
Supplement: Supplementary file 5 — Description of additional supplementary items [file 42003_2019_600_MOESM5_ESM.docx]

Description of additional Supplementary Files

Supplementary Data

Source data underlying the graphs and charts.
